# In-Depth Characterization of Live Vaccines Used in Europe for Oral Rabies Vaccination of Wildlife

**DOI:** 10.1371/journal.pone.0141537

**Published:** 2015-10-28

**Authors:** Florence Cliquet, Evelyne Picard-Meyer, Miroslav Mojzis, Zuzana Dirbakova, Zita Muizniece, Ingrida Jaceviciene, Franco Mutinelli, Marta Matulova, Jitka Frolichova, Ivan Rychlik, Vladimir Celer

**Affiliations:** 1 ANSES, Technopôle agricole et vétérinaire, Domaine de Pixérécourt, Malzéville, France; 2 State Veterinary and Food Institute Zvolen, Zvolen, Slovak Republic; 3 Institute of Food Safety, Animal Health and Environment BIOR, Riga, Latvia; 4 National Food and Veterinary Risk Assessment Institute of Lithuania, J. Kairiukscio, Vilnius, Lithuania; 5 University of Applied Sciences Faculty of Agrotechnologies, Vilnius district, Lithuania; 6 Istituto Zooprofilattico Sperimentale delle Venezie, Viale dell'Università 10, Legnaro (Padova), Italy; 7 Veterinary Research Institute, Hudcova 70, Brno, Czech Republic; 8 University of Veterinary and Pharmaceutical Sciences Brno, Department of Infectious Diseases and Microbiology, Brno, Czech Republic; Thomas Jefferson University, UNITED STATES

## Abstract

Although rabies incidence has fallen sharply over the past decades in Europe, the disease is still present in Eastern Europe. Oral rabies immunization of wild animal rabies has been shown to be the most effective method for the control and elimination of rabies. All rabies vaccines used in Europe are modified live virus vaccines based on the Street Alabama Dufferin (SAD) strain isolated from a naturally-infected dog in 1935. Because of the potential safety risk of a live virus which could revert to virulence, the genetic composition of three commercial attenuated live rabies vaccines was investigated in two independent laboratories using next genome sequencing. This study is the first one reporting on the diversity of variants in oral rabies vaccines as well as the presence of a mix of at least two different variants in all tested batches. The results demonstrate the need for vaccine producers to use new robust methodologies in the context of their routine vaccine quality controls prior to market release.

## Introduction

Rabies, one of the oldest recognized zoonotic diseases, is caused by negative-sense single-stranded RNA viruses of the genus *Lyssavirus* within the family *Rhabdoviridae*. Of the 14 recognized *Lyssavirus* species known to date [1], rabies virus (RABV) is distributed worldwide among mammalian hosts comprising various carnivores (mainly dogs, foxes and raccoons) and bats. Although rabies can be prevented by vaccination, this virus is still responsible for approximately 55,000 human deaths each year (http://who.int/rabies/en/). While in most developing countries, dogs are the major reservoir and transmitters of rabies, in developed countries wild animals play an important role in maintenance of the infection cycle [2].

Oral immunization of wild animals has been shown as the most effective method to control and eliminate rabies [3]. In this regard, significant progress was made in the development of oral rabies vaccines for the control of vulpine rabies. Three types of attenuated rabies vaccines are commonly used in Europe during oral vaccination campaigns for wild animals (namely the red fox and the raccoon dog). These are: the SAD Bern vaccine derived from the Street Alabama Dufferin (SAD) strain isolated from a naturally-infected dog in the USA in 1935 by passages on various cell cultures, SAD B19 derived by adaptation of the SAD Bern strain passaged in mice brain on cloned BSR cells, and SAG2 vaccine selected from the SAD Bern strain after two selective mutations of the Arginine 333 codon [4]. As a result of the mass oral vaccination campaigns on wildlife using attenuated live vaccines (e.g. SAD Bern, SAD B19 and SAG2) as well as a vaccinia-rabies glycoprotein recombinant virus vaccine (V-RG) in baits using aerial delivery methods, most Western and Central European countries, and more recently Northern European countries, have become rabies-free, with a total of 12 European countries [5].

Like other RNA viruses, RABV displays a high mutation rate due to the low fidelity of viral polymerase and heterogeneous RABV populations, often referred to as viral quasispecies [6]. The highly variable mixture of closely related genomes within a given host allows the viral population to rapidly adapt to dynamic environments [7]. Recent advances in nucleic acid sequencing technologies, referred to as “next-generation sequencing” (NGS) represent a true revolution in this area and open new perspectives for research and diagnostic applications [8], including clinical virology. Ultra-deep sequencing of intra-host rabies virus populations demonstrated that the newest NGS technology was useful for characterizing viral populations and may provide insight into ancestral genomes and the role of rare variants in viral emergence [9].

Attenuated live vaccines produced by repeated laboratory subpassages may represent safety issues because of their possible reversion to virulence. We previously showed that a live rabies vaccine widely used in Europe for wildlife oral vaccination showed higher similarity to the strains belonging to the SAD B19 strains than to the SAD Bern vaccines [4]. It therefore appeared necessary to carry out similar investigations on all the attenuated vaccines used in Europe for oral wildlife rabies vaccination (ORV), since inconsistencies in the vaccine composition of certain rabies vaccines were previously confirmed using standard Sanger sequencing [10]. Such studies may reveal the potential reversion to a virulent form and the possible identification of shifts in virus populations during the vaccine manufacturing process. Furthermore, recent developments in next genome sequencing now provide a simple tool for addressing this issue. In this study, we therefore characterized the genetic composition of three commercial attenuated live rabies vaccines as previously proposed [4] and as recommended by the competent regulatory authorities. We discovered that no single vaccine came from a single viral clone. In fact, all the vaccines were a mix of at least two different variants.

## Material and Methods

### 1.Viral samples

Three live attenuated rabies virus vaccines commercially available in Europe, which we called Vaccine A (batch A1, A2, A3), Vaccine B (B1, B2, B3) and Vaccine C (C1, C2, C3), were characterized in this study by next generation sequencing. The sequencing was performed in two independent laboratories which received blind-coded samples. All nine vaccine batches were collected from Rabies National Reference Laboratories in Lithuania (batches A1, A2, B1), Latvia (batches B2, B3, A3) and Italy (batches C1, C2, C3) in 2013. For vaccines A and C, the liquid suspension was collected from 15 plastic blisters and pooled, and for Vaccine B 8 baits were used. Blind-coded aliquots were distributed to laboratories for 454 pyrosequencing and Illumina sequencing assays using dry ice and triple packaging.

### 2.454 pyrosequencing

Viral RNA was extracted with the NucleoSpin® RNA kit (Macherey-Nagel) as per the manufacturer’s instructions and stored at –70°C until use. The first half of the rabies genome (positions 0–6433) was reverse transcribed from 1 μg of RNA using the Transcriptor First Strand cDNA Synthesis Kit (Roche) with a mixture of all 4 reverse primers listed in Table 1. One μL of cDNA was used as a template for PCR using Q5^®^ High-Fidelity DNA Polymerase (New England BioLabs). The PCRs were designed so that four partially-overlapping fragments with 4 pairs of primers were produced. For the list of primers, see Table 1. The four PCR products obtained for each vaccine batch were pooled, fragmented and ligated with adaptors with different sequence modifications (bar codes) using the GS Rapid Library Prep Kit and GS Rapid Library MID Adaptor Kit (Roche). This enabled the post-sequencing identification of each vaccine batch sequence after a single sequencing run. Following emulsion PCR using the GS Junior Titanium Lib-L emPCR Kit, the pyrosequencing was performed using the GS Junior Titanium Sequencing Kit according to the instructions of the manufacturer and the 454 GS Junior sequencer (Roche). Data were analyzed by the Reference Mapper included in the 454 GS Junior software using the SAD Bern genomic sequence as a reference (GenBank accession number EF206708).

**Table 1:** Characteristics of primers used for the reverse transcription and PCR amplification of viral RNA prior to 454 pyrosequencing.

Primer	Primer 5' - 3'	Position	Orientation
Set1f	ACGCTTAACAACCAGATCA	1–19	For
Set1r	GGTTTCACCTGAGAAGTAGTC	1247–1268	Rev
Set4f	CGACTAAAGAGATCTCACATAC	1313–1334	For
Set4r	TAGGTAGAAGACACCGCTACTC	3855–3876	Rev
Set9f	AGCGGTGTCTTCTACCTACT	3859–3878	For
Set9r	CGTCTCTTCGAGTTGACTTACC	6414–6435	Rev
SAD5	CTGACGTAGCACTGGCAGATGA	1200–1221	For
SAD6	TGACTGGAACAGGAAGCTAGGATC	1775–1798	Rev

Abbreviations: For, forward; Rev, reverse.

### 3.Illumina whole genome sequencing

Total RNA was extracted using the NucleoSpin^®^ RNA kit (Macherey-Nagel) followed by purification using the RNEasy mini-column as per the manufacturer’s instructions (Qiagen). The RNAs were stored at –70°C. RNA-Seq library construction was performed using a commercial service (Vertis Biotech, Freising, Germany) after ribo-depletion of samples. Following RNA-Seq library construction, 2x100 bp paired-end sequencing was performed on the HiSeq-2000 instrument (Illumina, San Diego, CA). Final sequence analysis was performed using Integrative Genomics Viewer (IGV) software based on variant analysis. The mean percentage of SADB19/SAG2 mutations for the first half of rabies genome was performed by comparison with the SAD Bern genomic sequence (EF206708) which was used as a reference for all analysis.

## Results

### 1.Nucleotide variation determined by 454 pyrosequencing

**Fig 1:**
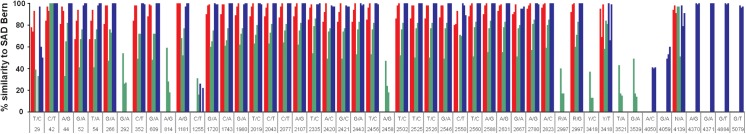
Strain mixtures present in 3 batches of Vaccine A (red columns), Vaccine B (blue columns) and Vaccine C (green columns). The figure represents the percentages of variants detected by 454 pyrosequencing. The SAD Bern genomic sequence (EF206708) was used as a reference. For example, three red columns show that the 3 Vaccine A batches differed from the SAD Bern reference sequences by more than 90%, i.e. the vaccine contained less than 10% of the SAD Bern strain. The description at the X axis shows the position relative to the referenced SAD Bern virus sequence (EF206708) and expected nucleotide present in the SAD Bern/observed nucleotide.

Only the high confidence variants identified for each vaccine batch by GS Reference Mapper, using SAD Bern sequence (EF206708) as a reference, were included. In the second selection step, we eliminated variants which were not present in all 3 batches of the same vaccine. Using this approach, 49 differences were identified among the vaccine sequences (S1 Table). Sequence coverage for the 9 analysed samples ranged from 13 to 428 with a median value of 151. The three tested batches of Vaccine A contained 15, 5.5 and 1.9% of SAD Bern strain (EF206708) and 85.0, 94.5 and 98.1% of SAD B19 strain (EF206709), respectively. The three Vaccine B batches contained on average 46.1, 28.3 and 22.3% of SAD Bern-like strain and 53.9, 71.7 and 77.7% of SAD B19 strain (EF206709), respectively. The vaccine strain population of the 3 batches of Vaccine C contained more than 97% of SAG2 strain (EF206719) which differed from the strains present in the remaining 2 vaccines by 4 specific mutations in positions 4370, 4371, 4884 and 5079 (Fig 1, Table 2 and S1 Table for additional detail).

**Table 2:** Analysis of the nine vaccine sequences using 454 pyrosequencing.

	Vaccine A			Vaccine B			Vaccine C		
Batch	A1	A2	A3	B1	B2	B3	C1	C2	C3
N	99^a^	98	88	73	53	68	99	100	100
P	97	100	88	80	61	72	100	100	100
M	96	99	89	82	53	77	100	99	99
Intergenicregion	93	100	84	80	51	75	99	98	98
Mean	94.5	98.1	85.0	77.7	53.9	71.7	99.2	97.2	97.8

Abbreviations: N, nucleoprotein; P, phosphoprotein; M, matrix protein.

a) Data show the mean percentage of SADB19/SAG2 mutations for the first half of rabies genome by comparison with the SAD Bern genomic sequence (EF206708) which was used as a reference for all analysis.

In addition to the major differences previously identified among the three vaccines [10], additional sequence variants were identified by pyrosequencing. Vaccine B contained variants present with an abundance of approximately 30% at positions 292, 814, 2458, 2997, 3418, 3521 and 3539 (Fig 1). These mutations collectively showed that the second viral strain present in Vaccine B in addition to SAD B19 was SAD VA1(pro) (EF206720), a strain closely related to the SAD Bern lineage [10]. Finally, Vaccine C was a mixture of two variants which were present in approximately the same proportion and differed from each other at positions 4050 and 4059 (Fig 1).

### 2.Nucleotide variation determined by Illumina sequencing

Full-length genome sequences were obtained for 8 samples only, since in one sample (Vaccine B, batch B2), the percentage of viral reads was lower than 0.002 of all the reads. Sequence coverage for this Vaccine B (batch 2) ranged from 4 to 208 with a median value of 32. Sequence coverage for the eight tested samples ranged from 10 to 2193 with a median value of 150 (S2 Table). In agreement with the pyrosequencing, the three batches of Vaccine A contained 13.8, 7.9 and 2.8% of the SAD Bern strain (EF206708) and 86.2, 92.1 and 97.2% of the SAD B19 strain (EF206709) respectively. The two Vaccine B batches contained an average of 18.6 and 18.2% of the SAD Bern like strain (EF206720) and 81.4 and 81.8% of the SAD B19 strain (EF206709) respectively. The vaccine strain population of the 3 batches of Vaccine C contained over 99% of the SAG2 strain (EF206719) (Table 3 and S2 Table).

**Table 3:** Analysis of the eight vaccine batches using whole genome sequencing with the Illumina MiSeq 2000 platform.

	Vaccine A			Vaccine B			Vaccine C		
Batch	A1	A2	A3	B1	B2	B3	C1	C2	C3
5'UTR	91^a^	100	78	90	NI^1^	86	100	100	100
N	94	98	88	80	NI^1^	80	100	100	100
P	89	98	90	68	NI^1^	71	99	100	100
M	87	90	82	77	NI^1^	77	99	99	100
Intergenic region	99	100	93	93	NI^1^	93	100	100	99
Mean	92.1	97.2	86.2	81.8	NI^1^	81.4	99.7	99.7	99.7

Abbreviations: N, nucleoprotein; P, phosphoprotein; M, matrix protein.

a) Data show the mean percentage of SADB19/SAG2 mutations for the first half of rabies genome by comparison with the SAD Bern genomic sequence (EF206708) which was used as a reference for all analysis.

#### Non interpretable

Vaccine C contained also a mixture of two variants at positions 4050 and 4059, which were present in approximately the same proportion (S2 Table). In addition to the differences identified by 454 pyrosequencing, an additional substitution was identified using whole genome Illumina sequencing. This mutation (constituted with an average of 69% of C and 31% of A) was detected in position 9876 (L gene) for the three tested batches and was specific to Vaccine C (data not shown).

## Discussion

Live rabies vaccines have to comply with international regulations in order to guarantee their identity and stability, as well as their efficacy and safety in target and non-target species [11–16]. More specifically, live rabies vaccines used in Europe should comply with the European Directorate for the Quality of Medicines regulations [11,12] requiring that several verifications be performed by the vaccine producer to avoid any mutations or contamination of the master seed virus strain by extraneous agents. The vaccine strain should be clearly identified using a suitable protocol in order to distinguish it from related strains, preferably through the use of full genome sequencing. To limit the risk of genetic changes, it is recommended that a maximum of five passages using the initial master seed virus be allowed for vaccine production [12,15,17].

Between 1978, the first year that ORV was carried out in Switzerland, and 2010, seven different SAD-derived oral rabies virus vaccines were used, including SAD Bern, SAD B19, SAG2, SAD P5/88, SAG1, SAD VA1, Vnukovo32 and one recombinant vaccine (V-RG) [18,19]. As a consequence, millions of baits have been distributed throughout Europe. All three oral rabies virus vaccines tested in our study were derived from the SAD Bern strain, either by selection on cloned BHK21 cells or by cultivation on cells under the selective pressure of monoclonal antibodies binding specifically to one of the two major antigenic sites (antigenic site III) of the rabies virus glycoprotein [20].

It was previously shown that the SAD B19 sequence (EF206709) differs from the SAD Bern sequence (EF206708) by more than 25 mutations. The sequence analysis of the three rabies virus strains showed that all tested Vaccine C batches were characterized in codon 333 of the G gene by the presence of the amino-acid Glutamine (100%), while sequences of the two other vaccines were characterized in this position by Arg_333_. The viral particles belonging to the SAD B19 clone, obtained by 454 pyrosequencing and by Illumina sequencing, ranged between 85% and 98.1% and between 86.2% and 92.1% respectively, in Vaccine A. The remaining virus particles corresponded to the SAD Bern clone (between 1.9% and 15% and between 7.9% and 13.8%, by 454 pyrosequencing and by Illumina sequencing, respectively). Vaccine B consisted of a mixture of two strains, SAD B19 and SAD VA1(pro), irrespectively of the method used. Between 53.9% and 77.7% and between 81.4% and 81.8% of the viral particles of this vaccine were from the SAD B19 sequence. The remaining virus particles (depending on the batch) was made up of SAD VA1(pro) (between 46.1% and 22.3% and between 18.2% and 18.6% by 454 pyrosequencing and by Illumina sequencing, respectively). This conclusion is based on the presence of five mutations in positions 292, 814, 2458, 2997 and 3418 specific to SAD VA1(pro). Vaccine C was found to contain more than 97% of SAG2 strain, irrespectively of the technique used. This vaccine was differentiated into two clones with variations in 4050 and 4059 positions, i.e. much less than the differences shown in Vaccines A and B. Although we propose two nearly equally represented clones, we cannot exclude that there might be four different clones, each equally represented and having A/C, A/A, C/G or C/A nucleotides in positions 4050 and 4059, respectively.

This is the first study to report on the diversity of variants in oral rabies vaccines using two independent NGS systems. Similar results were demonstrated regardless of the type of NGS used in the study. For addressing topics like composition of mixed viral populations, NGS is superior to the Sanger sequencing method that was performed earlier [10]. Whether our observations indicate that mutations accumulate during vaccine preparation similarly to *in vivo* situations in animal populations [9], or whether the producers have been continuously operating with mixed cultures, is difficult to interpret. However due to the number of differences in the viral clones found in Vaccines A and B, and since the ratios in all the individual mutations within a batch were quite similar, we can hypothesize that the producers had mixed cultures from the initial creation of their masters. Indeed, all attenuated rabies vaccines have been derived empirically from pathogenic clinical isolates by serial cultivation *in vitro* or *in vivo*, and our understanding of the genetic basis of their attenuation and the mechanism of this attenuation is still limited. Two variants were found for Vaccine C. This can be explained by the selective pressure of monoclonal antibodies to develop the vaccine. This likely resulted in original pure clone and the observed mutations in positions 4050 and 4059 could appear during subsequent propagation. This is at least indirectly supported by the fact that RNA viruses are commonly composed of highly diverse quasispecies populations that are generated during viral replication because of the high mutation rate of viral RNA polymerases. Few reports of Lyssavirus quasispecies which can confer high adaptability to the virus and enable cross-species transmission events, have been documented [6,21]. In 2013, for the first time, Borucki et al. [9] showed in a study of 44 unpassaged rabies viruses from the USA, the presence of rare variants within the studied viral population by applying deep-sequencing analysis to rabies virus samples from an outbreak in California in 2009.

Our sequencing data did not specifically address safety issues although this study showed a mix of strains for tested batches of two vaccines, although not in the same proportions. The issue raised here is probably not limited to oral rabies virus vaccines only, and may concern all conventional live virus vaccines for humans and animals. Since the vaccines currently used for rabies control in Europe were developed prior to the availability of the sequencing techniques, the vaccine producers were not able to detect those variants when they developed and released their master seed virus. Despite this, the results shown in the study justify the recommendations [13] for routine quality control of oral rabies vaccines using full genome analysis, next-generation sequencing representing an ideal tool for such controls.

Moreover, since RNA viruses are naturally composed of diverse quasispecies populations, a stability study of attenuated live rabies vaccines, coupled with the virus population study using NGS technology, could help to better understand live vaccine attenuation processes.

## Supporting Information

S1 Table Percentage of variants using 454 pyrosequencing in comparison to reference SAD Bern sequence, GenBank acc. n. EF206708, including coverage for each position expressed by number of reads per position. (XLS)

S2 Table Percentage of variants using Illumina RNA sequencing in comparison to reference SAD Bern sequence, GenBank acc. n. EF206708, including coverage for each position expressed by number of reads per position. (XLS)
